# Time for new guidelines in advanced healthcare: the mission of *The EPMA Journal *to promote an integrative view in predictive, preventive and personalized medicine

**DOI:** 10.1186/1878-5085-3-5

**Published:** 2012-03-28

**Authors:** Olga Golubnitschaja

**Affiliations:** 1 European Association for Predictive, Preventive & Personalised Medicine, Brussels, Belgium; 2 Department of Radiology, Rheinische Friedrich-Wilhelms-University of Bonn, Sigmund-Freud-Str. 25, D-53105 Bonn, Germany

**Keywords:** healthcare, strategy, advanced technologies, innovation, socioeconomic impacts, education

## Abstract

Changing long-held beliefs is never easy. As a consequence of the accumulating clinical data and knowledge about the epidemiology and pathological mechanisms of the most frequent causes of morbidity and mortality, we are currently reconsidering our view of the origins and progression of cardiovascular, oncologic and neurodegenerative diseases. Optimistic versus pessimistic prognosis for healthcare sector depends much on diagnostic, preventive and treatment approaches, which healthcare systems will preferably adopt in the near future. PPPM offers great promise for the future practice of medicine. We are pleased to announce this first open access volume of *The EPMA Journal *following its transfer to BioMed Central two years after publication of the first article in the journal.

## Why predictive, preventive and personalized medicine?

Predictive, preventive and personalized medicine (PPPM) offers great promise for the future practice of medicine. Essential components of this approach include well-organized population screening protocols utilizing novel diagnostic biomarkers of disease states, targeted prevention of common human pathologies, optimal treatment planning and personalized medicine thereby resulting in a substantial improvement in the quality of life. This approach also offers the advantage of delivering care at potentially reduced costs to the population at large, thereby addressing social and ethical issues related to access to and affordability of healthcare. A broad distribution and a routine clinical utilization of advanced technological approaches could enable a significant portion of the population to reach and exceed 100 years of age yet remaining vibrant and in excellent physical and mental health as actively contributing members of society. One of the central issues discussed is the screening for predisposition of healthy individuals to potential pathologies later in life. The groups at risk identified within the general population can be given a fair chance of a focused diagnosis with multidisciplinary expertise and well-timed preventive measures. The reliability of the blood-tests proposed, potential application to clinical routines, enormous economic and social impacts of the new generation of molecular- and nanotechnology-based diagnostic approaches - are all discussed in *The EPMA Journal*.

## Changing long-held beliefs is never easy

**Figure 1:**
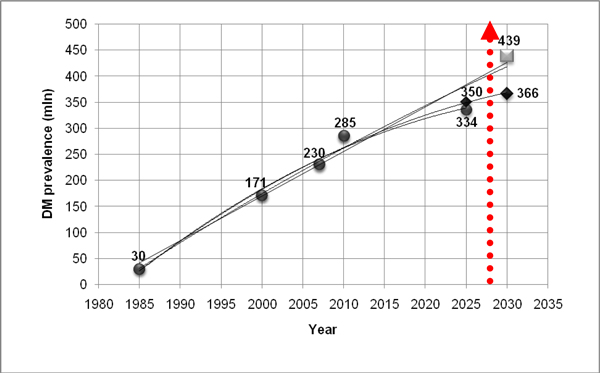
**Worldwide prevalence of diabetes mellitus with a more pessimistic prognosis for the next 3 decades: current literature updates **[1].

As a consequence of the accumulating clinical data and knowledge about the epidemiology and pathological mechanisms of the most frequent causes of morbidity and mortality, we are currently reconsidering our view of the origins and progression of cardiovascular, oncologic and neurodegenerative diseases. The majority of these pathologies are of chronic nature: they progress from precursor lesions over one or even several decades of life until a diagnosis is made that is often too late for effective therapeutic intervention. An excellent example is the epidemic scale of diabetes mellitus type 2 witnessed in the European Union. In most industrialized countries and countries with large populations, the permanently growing cohort of people with diabetes creates a serious healthcare problem and dramatic health economic burden. Estimates for diabetes prevalence in the years 2030 is half a billion patients worldwide [Figure 1].

Furthermore, the contemporary onset of the dominant type 2 diabetes has already been observed in subpopulations of teenagers [2]. Severe complications secondary to early onset of diabetes mellitus, such as retinopathy, nephropathy, silent ischemia, dementia, and cancer, could lead to collapsing healthcare systems.

Our message is that new guidelines should create the robust juristic and economic platform for advanced medical services utilizing the cost-effective models of risk assessment followed by tailored treatments focused on the precursor stages of chronic disease [3]. *The EPMA Journal *provides highly professional multidisciplinary insights into current "disease care" *versus *desirable "healthcare" mechanisms to promote the paradigm change from delayed interventional "disease care" to PPPM as the medicine of the future [4].

## Innovation by integrative view: PPPM as the "three-dimensional" vision in medical approaches

**Figure 2:**
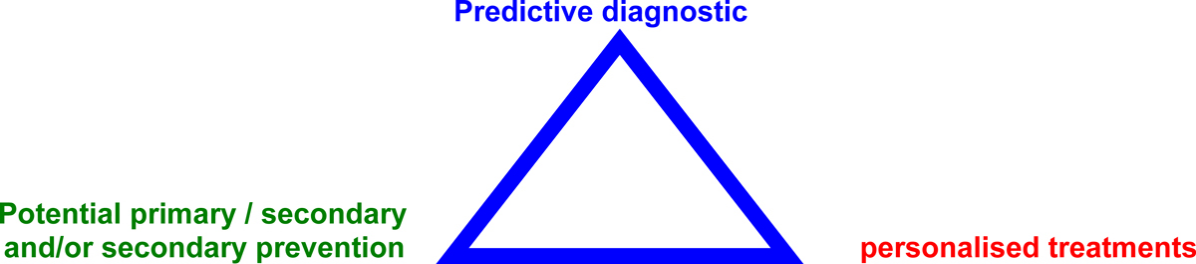
**"Three dimensional" vision in medical approaches**.

Optimistic *versus *pessimistic prognosis in future developments of the healthcare sector depends much on diagnostic, preventive and treatment approaches which healthcare will hopefully adopt in the near future [5]. Without innovation in healthcare, chronic disorders can cause such a dramatic level of burden that any performance of personalized medicine will not be feasible from an economic point of view. In contrast, effective utilization of advanced early/predictive diagnostics and targeted prevention could enable rapidly aging populations in Europe and elsewhere to be economically effective to society. Therefore, it is timely to consider the integrative medical approaches to advance healthcare utilizing the innovation by functional links within PPPM, such as predictive diagnostics as the basis for concomitant targeted prevention, patient profiling as the basis for individualized treatment algorithms and medical approaches tailored to the patient. In this context, *The EPMA Journal *promotes this "three-dimensional" vision in medical approaches, namely as outlined in [Figure 2].

## Specifications for article design, evaluation and acceptance in *The EPMA Journal*

There are some features of a paper design specific for the articles published with *The EPMA Journal *that are highly appreciated by readers, namely:

• Highly informative content that provides an overview of corresponding topics, including related multi- and inter-disciplinary aspects in a reader-friendly manner that makes articles particularly useful for professionals working in allied medical fields and non-medical braches.

• A reasonable combination of at least two aspects of the PPPM-concept; such as: predictive diagnostics followed by treatment design tailored to the patient; or innovative approach for population screening combined with concomitant recommendations for targeted prevention and individually created treatment algorithms.

• Discussion of the *status quo *(currents achievements & problems) overview followed by outlook for future trends and prognosis of developments

• A clear message in each publication supplied with a well-formulated PPPM-related title, a correspondingly structured abstract and conclusions including expert recommendations in the field

• Smart paper-design characterized by reader-friendly subheadings of paragraphs for ease of navigation

• A good balance between clinical aspects and technological solutions, *status quo *and perspectives, text and illustrative materials

• Overall excellent scientific and technological level of publications that undergo an additional evaluation by the Editorial Board (more than 50 experts from more than 30 countries worldwide) for consideration in "*The EPMA Journal Award*" for distinguished articles presented at the regular EPMA world congresses.

## Educational value of *The EPMA Journal*

*The EPMA Journal *is for everyone: on reading the journal, readers can learn a new philosophy in medicine, novel trends in healthcare and biomedical education. The journal provides important information for individuals of various professional and scientific backgrounds. Professionals can consider the general concept of multidisciplinary approaches and learn the mindset of related medical areas and other scientific branches. A partial list of those who will especially benefit from the information provided in the journal is as follows:

• professionals in conventional and molecular diagnostics, biomedicine, biotechnologies, ethics and economics, and the healthcare industry

• universities, research units, private and public hospitals

• patients and their family members

• international associations with healthcare-oriented scientific, research and public health-related activities/responsibilities

• political organizations and authorities active in the healthcare sector

**Figure 3:**
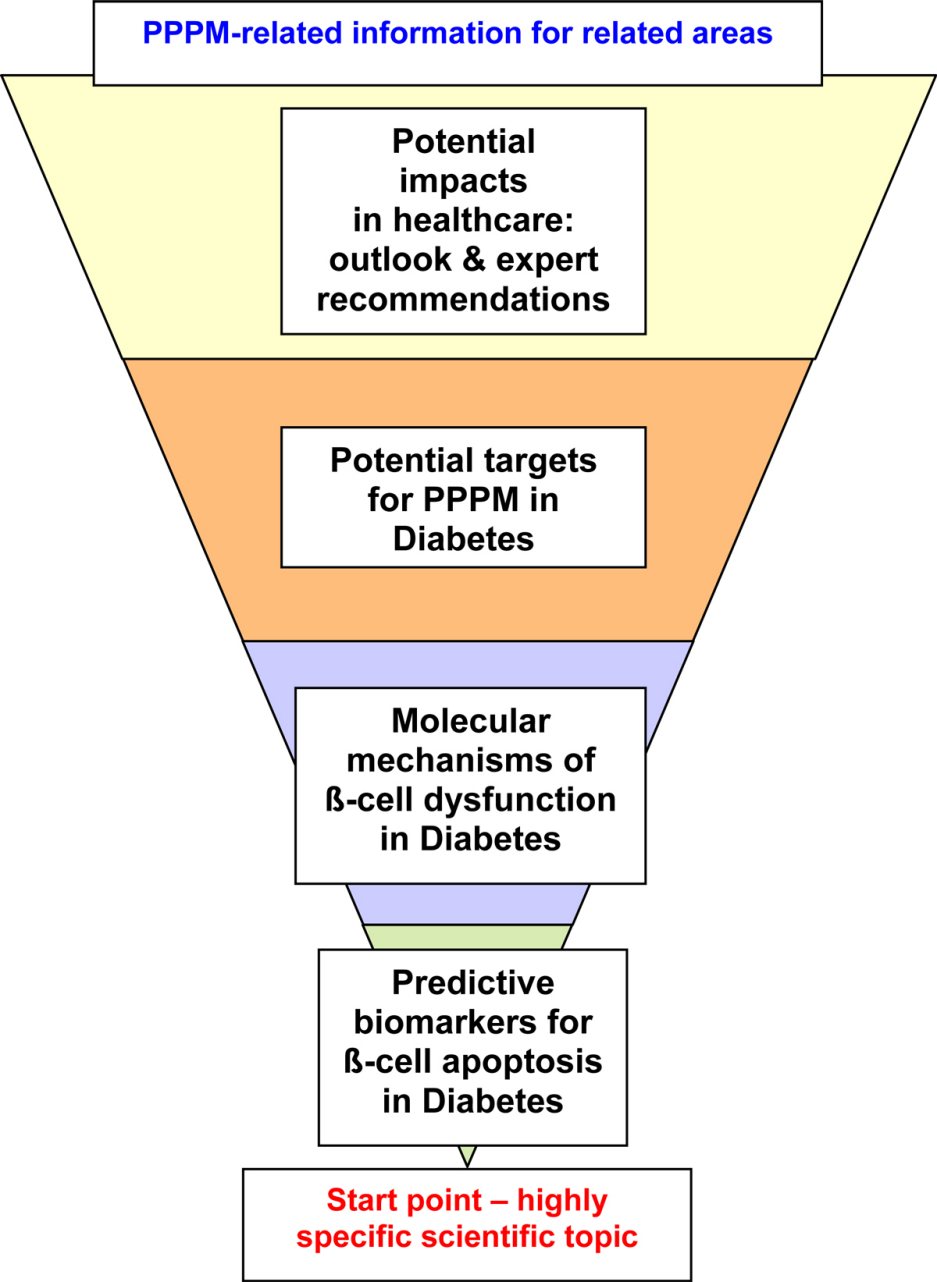
**The structure of informational pyramid characteristic for review articles in *The EPMA Journal*: an example is provided for diabetes research relevant for healthcare application under participation of several related areas**.

To reach the maximum educational impact, the review articles in *The EPMA Journal *are created according to the principle of the informational pyramid turned upside-down: highly specific topics that individual professional groups work on (the pyramid-tip) are brought into more generalized scientific context and, further, correlated with concrete tasks to be performed in the healthcare sector. Consequently, PPPM-related aspects potentially interesting for corresponding border areas and giving an overview of other scientific branches. An example is provided in [Figure 3]. The authors are requested to provide the outlook and expert recommendations for further developments in the branch.

The mission of the European Coordinator in this field of PPPM is performed by the European Association for Predictive, Preventive and Personalized Medicine. The concepts of innovative European and international projects, which EPMA represents for further consideration at the EU Commission, the European Parliament, NIH/NCI are elaborated upon by the consortium of the world-leading professionals (not restricted to Europe).

**Figure 4:**
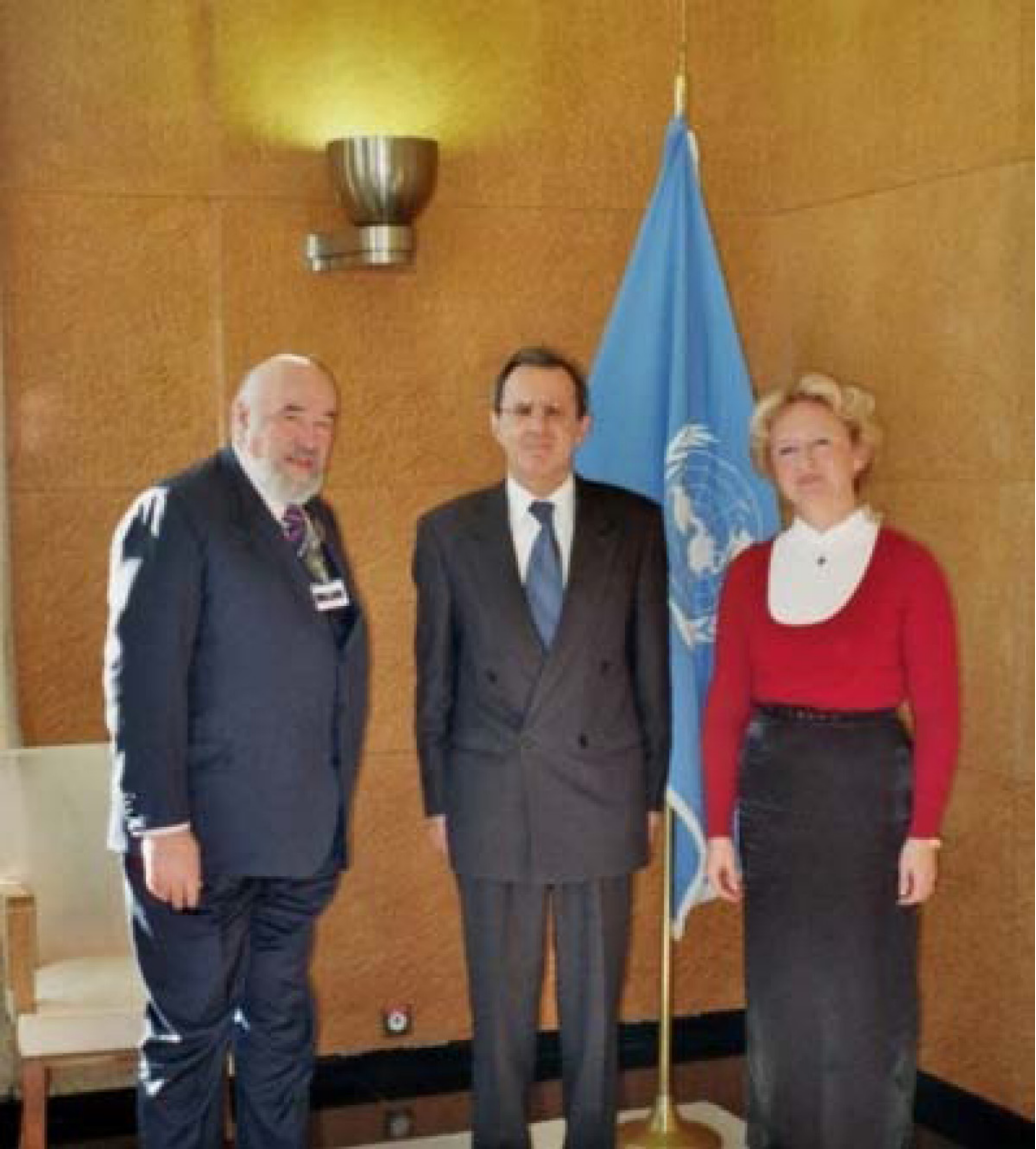
**EPMA goes global in consensus with United Nations: first meeting of the EPMA-Representatives with the Vice-Secretary General of UNO took place in Geneva on December 8th 2009**. From left to right: EPMA President - Vincenzo Costigliola, UNO Vice-Secretary General - Sergej Ordzhonikidze, EPMA Secretary General - Olga Golubnitschaja [2].

The EPMA Mission and Objectives in the field of predictive, preventive and personalized medicine have been introduced to UNO [Figure 4]. The participants of the meeting agreed that the paradigm change from curative to PPPM can only be achieved by coordinated measures focused on solving the accumulating problems in healthcare and the concomitant economic burden that societies across the globe are facing more and more. This is a new philosophy in healthcare and the platform for personalized patient's treatment considered as the medicine of future.

Practical application of innovative technologies in favour of predictive diagnostics, targeted preventive measures and personalized patient treatment in European healthcare and in global scale is the central idea of the Association. Through *The EPMA Journal*, the Association creates a professional forum to discuss most effective technologies and innovative approaches of predictive, preventive and personalized medicine.
